# YConvFormer: A Lightweight and Robust Transformer for Gearbox Fault Diagnosis with Time–Frequency Fusion

**DOI:** 10.3390/s25154862

**Published:** 2025-08-07

**Authors:** Yihang Peng, Jianjie Zhang, Songpeng Liu, Mingyang Zhang, Yichen Guo

**Affiliations:** 1 College of Mechanical Engineering, Xinjiang University, Urumqi 830017, China; 107552304306@stu.xju.edu.cn (Y.P.); 107552404347@stu.xju.edu.cn (S.L.); 107552404103@stu.xju.edu.cn (Y.G.); 2 College of Software, Xinjiang University, Urumqi 830091, China; 107552305021@stu.xju.edu.cn

**Keywords:** gearbox fault diagnosis, time–frequency fusion, axial decomposition, lightweight model, noise robustness

## Abstract

This paper addresses the core contradiction in fault diagnosis of gearboxes in heavy-duty equipment, where it is challenging to achieve both lightweight and robustness in dynamic industrial environments. Current diagnostic algorithms often struggle with balancing computational efficiency and diagnostic accuracy, particularly in noisy and variable operating conditions. Many existing methods either rely on complex architectures that are computationally expensive or oversimplified models that lack robustness to environmental interference. A novel, lightweight, and robust diagnostic network, YConvFormer, is proposed. Firstly, a time–frequency joint input channel is introduced, which integrates time-domain waveforms and frequency-domain spectrums at the input layer. It incorporates an Efficient Channel Attention mechanism with dynamic weighting to filter noise in specific frequency bands, suppressing high-frequency noise and enhancing the complementary relationship between time–frequency features. Secondly, an axial-enhanced broadcast attention mechanism is proposed. It models long-range temporal dependencies through spatial axial modeling, expanding the receptive field of shock features, while channel axial reinforcement strengthens the interaction of harmonics across frequency bands. This mechanism refines temporal modeling with minimal computation. Finally, the YConvFormer lightweight architecture is proposed, which combines shallow feature processing with global–local modeling, significantly reducing computational load. The experimental results on the XJTU and SEU gearbox datasets show that the proposed method improves the average accuracy by 6.55% and 19.58%, respectively, compared to the best baseline model, LiteFormer.

## 1. Introduction

The heavy equipment manufacturing industry is a vital pillar of the industrial sector and a strategic industry closely related to national security. In this field, the operational safety of machinery directly affects production efficiency and safety. Studies have shown that about 70% of failures in rotating machinery are related to rotors [1,2], and as a key component in the power transmission system, gearboxes are prone to various types of faults due to their high-intensity operational tasks, resulting in increased maintenance costs, property damage, and even safety accidents [3,4,5]. Therefore, conducting intelligent fault diagnosis research for gearboxes is of great significance to ensure production efficiency and equipment reliability [6].

With the increasing complexity of industrial equipment, fault diagnosis technology has evolved from relying on human expertise to being driven by artificial intelligence [7]. Traditional methods mainly rely on signal processing, statistical analysis, and shallow models. Although they achieved certain success in early applications, their limitations gradually became apparent in dynamic, high-noise industrial environments [8,9]. Moreover, due to the need for specialized knowledge and the difficulty in capturing subtle fault features, these methods often fail to meet the demands of accurate identification and large-scale data processing [10,11].

In the AI-driven fault diagnosis field, the technology is mainly divided into two major approaches: methods based on traditional machine learning algorithms and those based on deep learning frameworks, both of which constitute the main architecture of the current intelligent fault diagnosis system [12,13]. Machine learning techniques, by analyzing large-scale datasets, can effectively mine hidden fault features and achieve high-precision, high-efficiency equipment status recognition. Numerous studies have confirmed the feasibility of machine learning in automated fault diagnosis and its widespread application in industrial equipment health monitoring [14,15]. In recent years, scholars have optimized feature extraction by introducing techniques like time-domain statistical calculations and frequency-domain energy spectrum analysis, significantly improving the diagnostic accuracy and robustness of traditional machine learning models [16,17,18,19]. However, such methods typically use shallow model architectures, which have the inherent limitation of limited feature representation ability. In dynamic industrial environments, classification models built on relatively closed assumptions struggle to capture nonlinear associations in complex systems, making their performance bottlenecks particularly apparent in open environments with complex operating conditions and multi-source interference.

In recent years, deep learning technology has brought revolutionary breakthroughs to fault diagnosis. Convolutional Neural Networks (CNNs), with their outstanding ability to extract local features, have become the mainstream method [20]. For one-dimensional vibration signals, researchers have enhanced the model’s ability to identify subtle fault features by improving convolutional kernel designs, such as multi-scale convolutions and adaptive pooling, as well as introducing attention mechanisms [21,22,23,24]. For two-dimensional time–frequency map inputs, lightweight architectures [25] and hybrid network structures [26] have become mainstream solutions, effectively balancing computational efficiency and diagnostic accuracy. Representative works include the following: He et al. [27] proposed ResNet18, which significantly improved the training efficiency and classification accuracy of deep networks by optimizing residual mapping and aggregating hierarchical features; Liu et al. [28] introduced MK-ResNet, which improves the model’s diagnostic robustness under non-stationary conditions by relying on multi-scale feature fusion and residual learning mechanisms. However, the local receptive field characteristic of CNNs limits their ability to model cross-period correlation features in long-sequence signals, and pooling operations may lead to the loss of subtle fault information, which is an inherent limitation [29].

To overcome these limitations, researchers began to introduce Transformer models. The core self-attention mechanism of Transformers can globally capture the temporal dependencies between signals, offering two main advantages: it models long-range dependencies by computing attention weights across all time points, and it adaptively focuses on key temporal segments that are sensitive to faults [30,31,32]. Representative works include the following: Fang et al. [33] proposed CLFormer, which demonstrated strong robustness under small-sample conditions; Han et al. [34] proposed ConvFormer-NSE, which achieved excellent diagnostic performance in noisy environments by combining sparse self-attention mechanisms; Yan et al. [35] developed LiConvFormer, which used separable multi-scale convolutions and Broadcast Self-Attention to significantly reduce the computational complexity of hybrid architectures; and Sun et al. [36] proposed the lightweight and efficient Transformer model LiteFormer, which innovatively replaces the standard self-attention mechanism with convolutional layers and integrates depthwise separable convolutions to reduce computational load, significantly improving diagnostic real-time performance. LiteFormer also generates lightweight discriminative feature vectors through cross-layer feature recalibration modules and global average pooling (GAP) layers.

At the same time, some networks supporting parallel inputs have played an increasingly important role in fault diagnosis. Parallel-structured network models reduce redundant parameters through symmetric weight sharing or structural constraints, aligning with the concept of lightweight design. DCNN [37] processes time-domain and frequency-domain information in parallel through a symmetric dual-channel design. Pan et al. [38] proposed a dual-branch architecture that decomposes signals into low-frequency structures and high-frequency details, extracting global structural features with shallow networks and local detail features with deep networks, which are jointly optimized through a fusion module. Lin et al. [39] introduced a Convolution and Cross-Fusion Transformer (CCFT), which strengthens the ability to capture subtle fault features by parallel processing time-domain vibration signals and frequency-domain time–frequency map modes, combined with a multi-head self-attention mechanism. Cross-modal cross-attention fusion and hierarchical residual connections are used to generate discriminative hybrid feature vectors. Snyder et al. [40] proposed a Dual-Head Ensemble Transformer (DHET) for bearing fault diagnosis, combining a 1D Transformer for time-domain signals and a 2D Vision Transformer (ViT) for spectrogram analysis. The model uses self-attention to capture long-range dependencies and global correlations, fusing features from both domains for high accuracy.

Existing dual-modal network models (such as DCNN and CCFT) effectively improve diagnostic accuracy by processing dual-modal signals in parallel. However, three emerging CNN–Transformer hybrid architectures (CLFormer, ConvFormer-NSE, and LiteFormer), although showing significant advantages in mechanical fault diagnosis, still face the following key limitations:

1. Limitations of Time-Domain Signals: Time-domain signals can only represent temporal amplitude changes, making it difficult to directly reveal key frequency-domain features such as inherent device frequencies and resonance points. This results in insufficient capability to distinguish between atypical faults that exhibit similar time-domain patterns. Additionally, time-domain signals are easily affected by high-frequency noise, severely limiting the model’s noise robustness under single-modal input conditions.
2. Efficiency Bottleneck of Attention Mechanisms: Single-head attention is limited by its representation in a single subspace and struggles to adequately model complex temporal dependencies. Although Multi-Head Attention enhances feature diversity through parallel weighted matrices, its computational complexity increases quadratically with the sequence length, leading to a significant computational burden.
3. Inefficiency of Dual-Channel Architectures (Time-Domain and Frequency-Domain Branches): Dual-channel architectures, such as DCNN, independently process different modalities. While convolutional layers stacked in these architectures enhance feature extraction capabilities, the model efficiency is reduced due to redundant parameter computations.

It is noteworthy that although LiConvFormer, proposed by Yan et al., offers lightweight advantages, it still faces issues such as insufficient feature extraction and weak noise robustness during time-domain training. To address these challenges, researchers have proposed targeted optimization schemes: Wang et al. [41] introduced the Efficient Channel Attention (ECA) module, which aggregates global channel-level information through global average pooling (GAP) to preserve complete feature representation; Wang et al. [42] developed Axial Attention, which independently applies attention mechanisms along the axial components of multi-dimensional inputs (such as rows and columns), significantly reducing computational complexity while enhancing global modeling capabilities.

Based on the aforementioned research, this paper proposes a novel diagnostic network that integrates the ECA-enhanced time–frequency input and axial-enhanced attention mechanisms. The core innovative contributions are as follows:

1. ECA-Enhanced Time–Frequency Joint Input Channel: A pioneering approach is introduced, using a pre-FFT module to construct a hybrid time–frequency representation. After the ConvBNReLU layer, an ECA module is embedded, which dynamically weights and selects frequency band features with strong noise resistance. This effectively suppresses high-frequency noise interference in the time domain and enhances the complementary relationship between time-domain waveforms and frequency spectrum energy.
2. Axial-Enhanced Broadcast Attention Mechanism: An innovative axial decomposition strategy is introduced to improve the broadcast attention mechanism. Spatial axial convolution models long-range temporal dependencies, expanding the receptive field of shock features, while channel axial convolution establishes cross-frequency band associations to strengthen the interaction of harmonic components. This design exchanges minimal computational overhead for an expanded receptive field, significantly improving the fine-grained temporal modeling capability.
3. Lightweight Robust Collaborative Optimization Architecture: Unlike existing Transformer, CNN, and parallel models, this method is the first to fuse time–frequency information (empowered by ECA) at the input channel level. After shallow feature processing, the model completes global–local modeling through collaborative Axial Attention and convolutional layers. Experiments show that this architecture significantly reduces computational resource consumption while surpassing the latest benchmark methods in noise robustness, effectively solving the core contradiction between lightweight design and robustness.

## 2. Related Theories and Technologies

### 2.1. Fast Fourier Transform

The Fast Fourier Transform (FFT) is an efficient algorithm for computing the Discrete Fourier Transform (DFT) and its inverse transform. Its core value lies in significantly reducing the direct computation complexity of DFT from O(N^2^) to O(NlogN), greatly improving signal processing efficiency. This technology has been deeply applied in fields such as communication systems, medical diagnostics, and vibration analysis, playing an irreplaceable role in fault detection, spectral analysis, and real-time systems [43]. Gou et al. developed a three-phase current spectrum analysis technique based on the FFT algorithm [44]. This method applies FFT to current signals to generate a spectrum that represents the energy distribution of different frequency components. Consequently, the most fault-sensitive characteristic frequencies are selected as model inputs, significantly improving diagnostic efficiency. Meanwhile, Zhu and Peng conducted research on the issues of wind power abandonment and transmission interruptions caused by short circuits in power lines in wind farms under the application of doubly fed asynchronous generators. Their approach uses FFT to extract the fundamental wave amplitude of the zero-sequence current and constructs a multi-dimensional feature set for more accurate fault location [45].

From the perspective of frequency-domain symmetry and phase rotation, the efficiency of FFT stems from the hierarchical decomposition of the periodicity of the rotation factor. By decomposing the DFT into a weighted combination of two interlaced subsequences and introducing phase correction terms to establish recursive relations, the original DFT expression is rewritten into a block summation form. Therefore, we obtain the following results:

(1) $$X\left(k\right)=\sum_{m=0}^{\frac{N}{2}-1}\;x_{L}\left(m\right)\omega_{N}^{mk}+\sum_{m=\frac{N}{2}}^{N-1}\;x_{H}\left(m\right)\omega_{N}^{mk}$$

where $x_{L}$ represents the low-frequency component, and $x_{H}$ represents the high-frequency component. Let $m^{\prime }=m-\frac{N}{2}$, and after substituting the variable, the expression can be transformed into the following:

(2) $$\begin{array}{c} X\left(k\right)=\sum_{m^{\prime }=0}^{\frac{N}{2}-1}\left[x_{L}(m^{\prime })+(-1{)}^{k}x_{H}(m^{\prime }+\frac{N}{2})\right]\omega_{N}^{m^{\prime }k}\\ =\sum_{m^{\prime }=0}^{\frac{N}{2}-1}\left[x_{L}\left(m^{\prime }\right)+\omega_{\frac{N}{2}}^{k}x_{H}\left(m^{\prime }\right)\right]\omega_{N}^{m^{\prime }k} \end{array}$$

where $\omega_{\frac{N}{2}}=e^{-j\frac{2\pi }{\frac{N}{2}}}$ and the phase factor $\theta (k)=\omega_{N}^{\frac{k}{2}}$; the expression can be written as follows:

(3) $$X\left(2k\right)=X_{L}\left(k\right)+\theta \left(k\right)X_{H}\left(k\right)\;X\left(2k+1\right)=X_{L}\left(k\right)-\theta \left(k\right)X_{H}\left(k\right)$$

The FFT’s butterfly computation structure reduces the computational load to $\frac{1}{2}{log}_{2}N$ of the original DFT, with only *N* complex multiplications and additions required per layer. This decomposition strategy eliminates redundant calculations, showcasing significant advantages in real-time systems such as signal processing.

### 2.2. Multi-Head Attention Mechanism

**Figure 1:**
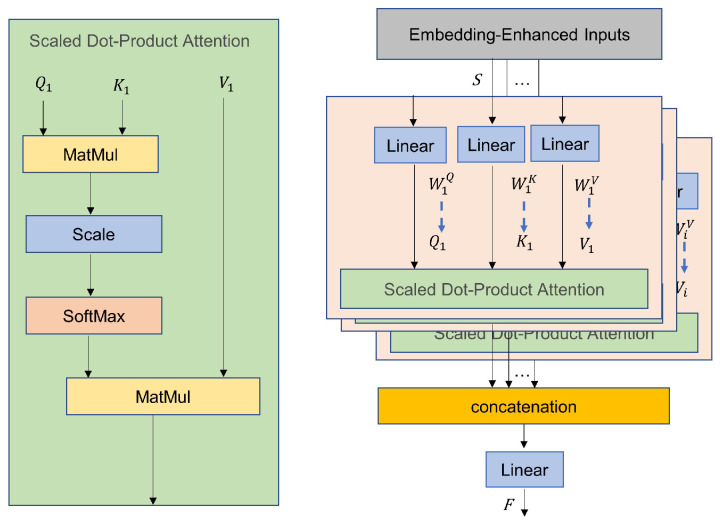
MHA structure.

The Multi-Head Attention (MHA) mechanism is a core architecture in deep learning models that enhances the model’s ability to capture diverse contextual information by parallelizing feature learning. The core idea is to split the input features into multiple subspaces (referred to as “heads”), each of which learns different patterns of representation, and then fuse the results [46]. Its structure is shown in Figure 1. First, the input feature vectors are split into multiple independent submodules, or “heads,” via linear projection. Each head learns the correlation patterns of the input data in different subspaces. Then, each head independently generates query (Query), key (Key), and value (Value) matrices. Attention weights are calculated using the scaled dot product similarity, represented by the following formula:

(4) $$H_{i}=Softmax\left(\frac{S_{i}W_{i}^{Q}(S_{i}W_{i}^{K}{)}^{T}}{\sqrt{d}}\right)S_{i}W_{i}^{V}$$

where $H_{i}$ represents the attention weights for the *i*-th head, $S_{i}\in R^{B\times C\times L}$ is the input for the *i*-th head, with B as the batch size, *C* as the number of channels, and *L* as the sequence length. $W_{i}^{Q}$, $W_{i}^{K}$, $W_{i}^{V}$ represent the projection matrices for the *i*-th head. $SW_{i}^{Q}(SW_{i}^{K}{)}^{T}$ calculates the matching score between queries and keys, and SoftMax converts the similarity into a probability distribution with weights summing to 1. $SW_{i}^{V}$ represents the weighted sum of the value matrix according to the attention weights. d is the dimension of the vector, which prevents the dot product results from becoming excessively large in high-dimensional spaces, causing vanishing gradients in the SoftMax. The output of the Multi-Head Attention mechanism is then obtained by concatenating the outputs of all heads and applying a final linear projection:

(5) $$F=Concat\left(H_{1},H_{2},\ldots ,H_{i}\right)W^{0}$$

where $H_{1},H_{2},\ldots ,H_{i}$ are the outputs of all attention heads, and $W^{0}$ is the output projection matrix that maps the concatenated features back to the original model dimension.

Based on the above computation process, the parameters and FLOPs of the Multi-Head Attention (MHA) block can be expressed as follows:

(6) $${Params}_{MHA}=4d^{2}\;{FLOPs}_{MHA}=4nd^{2}+2n^{2}d+n^{2}h$$

where $4nd^{2}$ is the FLOPs for the input and output projections, $2n^{2}d$ is the FLOPs for each head’s Q and K dot product computation as well as the weighted summation of the value matrix and attention weights, and $n^{2}h$ is the FLOPs for the multi-head SoftMax operation, where h is the number of attention heads.

## 3. Proposed Model

### 3.1. Time–Frequency ECA-Enhanced Input

**Figure 2:**
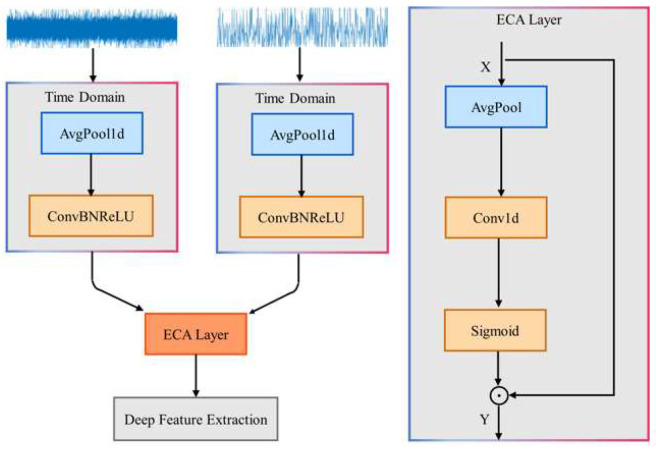
TFE input.

Traditional single-domain feature processing paradigms often suffer from the separation of time–frequency information, making it difficult to efficiently process noisy and complex signals. In this paper, we propose the Time–Frequency ECA (TFE) module, which uses time–frequency dual-branch feature encoding and ECA channel dynamic weighting to achieve complementary time–frequency information and adaptive feature enhancement. The working process of this module is shown in Figure 2.

First, the module takes both time-domain waveforms and frequency-domain spectra as dual inputs. Time-domain and frequency-domain information are naturally complementary, but they need to be jointly modeled to unlock their full potential. Then, independent branches are set for each input to perform noise reduction and redundancy elimination, followed by rough feature extraction. The parallel structure of the dual branches preserves both the temporal continuity and frequency-domain distribution. At the same time, the ConvBNReLU operations provide robust and discriminative dual-domain features for subsequent attention weighting. Finally, ECA treats the time–frequency dual-branch features as a joint input along the channel dimension, using channel weighting to enhance the key time-domain and frequency-domain features collaboratively, breaking down the information barrier between the time and frequency domains. Compared to traditional convolutions with fixed weights, the dynamic weighting in ECA makes the module more robust in noisy and time-varying scenarios. The following Equation (7) describes the ECA module:

(7) $$z=\frac{1}{L}\sum_{i=1}^{L}\;X_{i}\;F=\sigma \left(Conv1D_{k}\left(z^{⊤}\right)\right)⊙X$$

where $X\in R^{B\times C\times L}$ is the input to the ECA module, $X_{i}$ represents the feature vector at the *i*-th spatial position of the input features, and $z\in R^{B\times C\times 1}$ is the output feature from global average pooling. $F\in R^{B\times C\times L}$ is the output of the ECA module. $\sigma \left(Conv1D_{k}\left(z^{⊤}\right)\right)$ generates the channel attention weights, and ⊙ represents broadcast multiplication, which recalibrates the features.

### 3.2. Axial Enhanced Broadcast Attention

**Figure 3:**
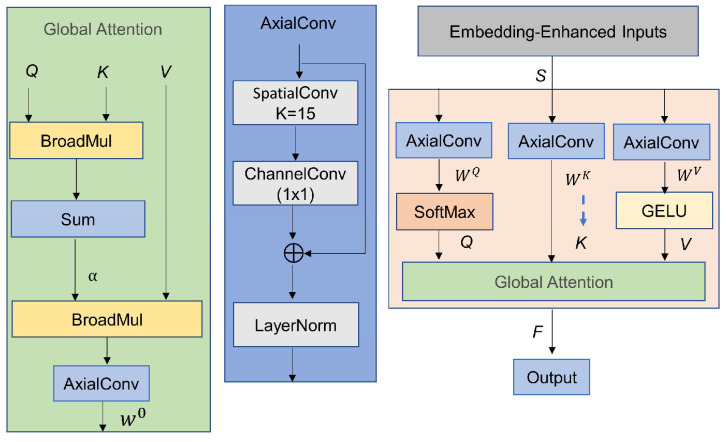
AEBA structure.

To address the computational bottleneck caused by the global dense matrix multiplication and SoftMax operation (both with time complexity of O(N^2^) in MHA), this study combines the dimensional decoupling idea from separable attention and the lightweight interaction characteristics of Broadcast Self-Attention (BSA) to propose the Axial Enhanced Broadcast Attention (AEBA) module (structure shown in Figure 3). AEBA introduces Axial Convolution (AxialConv) to decouple the spatiotemporal feature interactions. It significantly enhances the ability to capture long-range dependencies while maintaining the advantages of one-dimensional temporal modeling and lightweight global information propagation. The principle is as follows.

Axial Decomposition Feature Projection: Let the input signal S be enhanced with embedding, and use Axial Convolution (AxialConv) to generate Q, K, and V:

(8) $$Q=SoftMax\left(W^{Q}\cdot AxialConv\left(S\right)\right)\;K=W^{K}\cdot AxialConv(S)\;V=GELU\left(W^{V}\cdot AxialConv\left(S\right)\right)$$

where *S* is the input sequence enhanced with embedding, and AxialConv is the axial decomposition convolution, implemented through depthwise separable convolutions, channel convolutions, residual connections, and Layer Normalization to achieve decoupling of spatiotemporal features. *GELU* (Gaussian Error Linear Unit) is used to retain negative values to enhance the nonlinear expression.

Broadcast Attention Interaction: Q is broadcast to the K, enabling lightweight global information propagation. After summing along the temporal dimension, a global context vector α is generated:

(9) $$\alpha =\sum_{i=1}^{C}\;\left(Q_{i}⊙K_{i}\right)$$

where ⊙ denotes element-wise broadcast multiplication. When Q is a one-dimensional time score and K is a multi-channel key feature, Q is automatically expanded to match the dimensions of K. The resulting vectors are then multiplied element-wise, avoiding the high-dimensional matrix operations of traditional attention.

Global Context Fusion: The global context vector α is then broadcast to the value matrix V, where it is activated by GELU to enhance the fine-grained features with global information. Finally, Axial Convolution is applied to complete the feature projection and obtain the output F:

(10) $$F=AxialConv\left(\sum_{i=1}^{C}\;\left(\alpha ⊙V\right)\right)$$

This design exchanges minimal computational overhead for an expanded receptive field, significantly improving the fine-grained temporal modeling capability. According to the computational procedures described earlier, the Params and FLOPs for AEBA can be expressed as follows:

(11) $${Params}_{AEBA}=3C^{2}+4C+2(kC+C^{2}+3C)\;{Flops}_{AEBA}=N\left(5C^{2}+2kC\right)+3CN+N$$

where $3C^{2}+4C$ are the linear projection parameters, and $2(kC+C^{2}+3C)$ are the parameters for two Axial Convolutions. $N\left(5C^{2}+2kC\right)$ represents the computation cost for decompositions and linear projections, 3CN accounts for the broadcast operations, and N is the number of SoftMax single-dimensional exponential operations.

### 3.3. Overall Network Architecture

**Figure 4:**
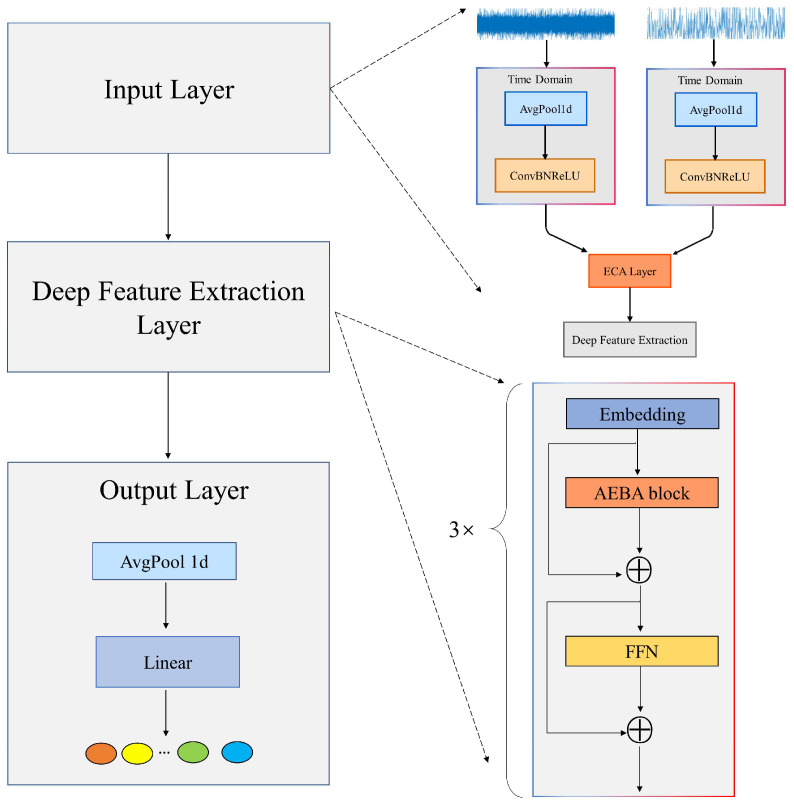
Network architecture diagram.

**Figure 5:**
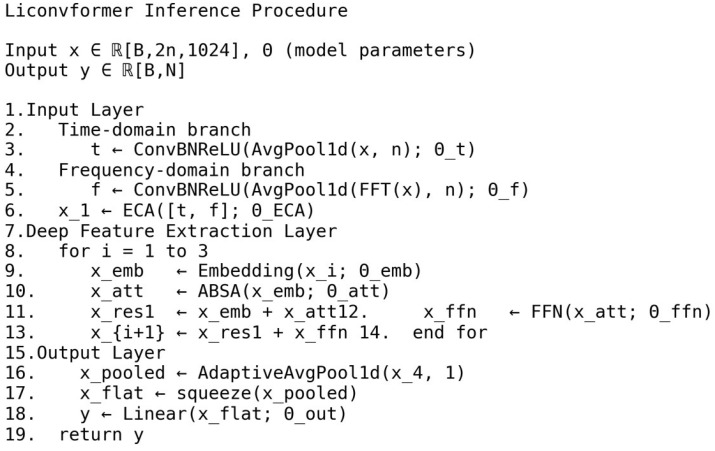
Proposed architecture pseudocode.

The proposed model adopts a hierarchical architecture comprising three main components: the signal input module, the Deep Feature Extraction Module, and the Output Classification Module, as shown in Figure 4; the corresponding pseudocode is presented in Figure 5.

In the input stage, the model simultaneously processes time-domain vibration signals and their corresponding frequency-domain spectra. First, both modalities undergo pooling operations to reduce dimensionality and suppress redundant noise. Then, 1D convolutional layers are applied to extract local features from adjacent sampling points, enabling initial representation learning. Next, the extracted features from both domains are concatenated to form a unified input. This combined representation is passed through an Efficient Channel Attention (ECA) layer, which generates dynamic channel-wise attention weights. Specifically, global average pooling (GAP) is first applied to the combined features to capture global statistical characteristics. These are then passed through a 1D convolution layer followed by a Sigmoid activation to produce attention scores. Finally, broadcast multiplication is used to recalibrate the features, adaptively enhancing key components in both the time and frequency domains—effectively breaking the information barrier associated with traditional single-domain processing.

In the Deep Feature Extraction Module, this module employs a three-stage progressive cascade design: the embedding layer maps the low-dimensional time–frequency features to a higher-dimensional space, expanding the model’s expressive capacity. The core AEBA module leverages axial decomposition-based attention mechanisms to model long-range temporal dependencies while maintaining computational efficiency. It also enables the network to focus on fault-sensitive components. A lightweight Feed-Forward Network (FFN), consisting of Layer Normalization and learnable residual connections, performs nonlinear feature transformations and ensures stable gradient flow. The three-stage structure enables progressively deeper feature refinement and iterative enhancement.

In the Output Classification Module, a global temporal pooling operation is applied to eliminate variations along the time dimension and distill a compact, high-quality feature vector. This vector is then passed through a fully connected layer that linearly maps the high-dimensional representation into a predefined fault category space, thereby achieving end-to-end identification from raw vibration signals to health states.

This architecture not only ensures strong feature representation capability but also significantly improves computational efficiency. Table 1 details the core configuration parameters and feature dimension changes for each component of the proposed network. The input signal length is fixed at 1024 data points, and B denotes the batch size.

### 3.4. Model Fault Diagnosis Framework

**Figure 6:**
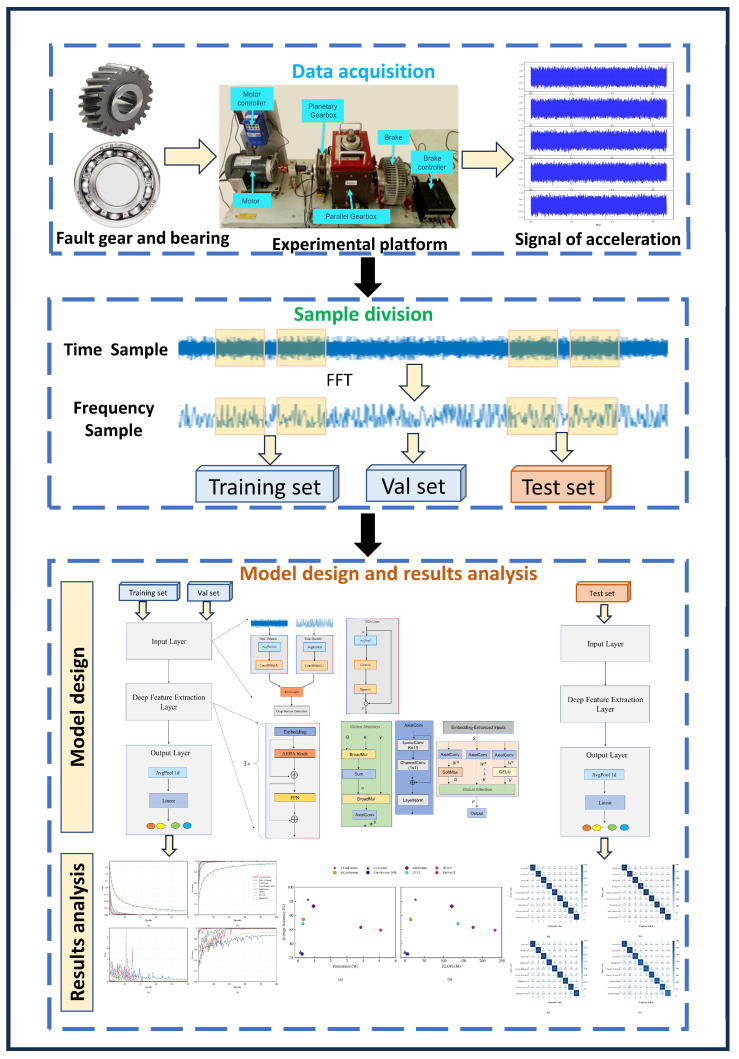
Overall process of the YConvFormer diagnostic model.

The complete workflow of data acquisition, preprocessing, model training, and evaluation is illustrated in Figure 6.

1. Acceleration vibration signals are collected from a composite experimental platform, which integrates faulty gears, bearings, motor controllers, and planetary gearboxes.
2. The raw time-domain signals undergo Fast Fourier Transform (FFT) to generate frequency-domain spectra. This creates a time–frequency dual-domain dataset. The continuous time-series data is split into independent sample units using a non-overlapping sliding window strategy. These samples are then divided into training, validation, and test sets according to a preset ratio. This division strategy ensures strict avoidance of information leakage between samples.
3. Model training and performance evaluation are conducted, followed by visualization analysis. The training and validation sets are input into the model to optimize the parameters while recording the learning trajectory. During the testing phase, the optimized model is used to identify fault types. The recognition accuracy is quantitatively analyzed using a confusion matrix, and qualitative evaluation of the diagnostic results is performed through feature visualization techniques. This enables multi-dimensional, comprehensive verification of the model’s performance.

## 4. Results and Discussion

This chapter systematically evaluates the performance of the proposed YConvFormer model in three aspects: lightweight design, noise robustness, and architectural innovation. Section 4.1 provides a detailed description of the experimental setup and seven comparison models. Section 4.2 uses the XJTU planetary gearbox dataset to validate the model’s diagnostic accuracy, computational efficiency, and feature discriminability in noisy environments. Section 4.3 tests the model’s generalization ability using the SEU gearbox dataset. Section 4.4 conducts ablation experiments to quantitatively analyze the contributions of the two core modules—TFE and AEBA—to performance improvement, revealing the effectiveness of the lightweight robust collaborative optimization mechanism.

### 4.1. Preparation

This study systematically compares the proposed YConvFormer method with seven benchmark models: the traditional CNN architecture; ResNet18; four state-of-the-art ConvFormer models: LiConvFormer (proposed in 2024), CLFormer (proposed in 2022), ConvFormer-NSE (proposed in 2023), and LiteFormer (proposed in 2024); and two dual-modal network models: DCNN and CCFT (proposed in 2024).

To assess the robustness of the proposed method in a real industrial noise environment and simulate potential distribution discrepancies between training and testing data in manufacturing scenarios, two different types of noise were intentionally injected into the test set [47]. Noise was added to the test dataset to simulate real-world disturbances, ensuring the model’s performance is robust under challenging conditions. The equation is as follows:

(12) $$x_{i}^{\prime }=\{\begin{array}{cc} x_{i}+\varepsilon , & \varepsilon ∼N\left(0,\lambda \right)\\ x_{i}\times \sigma , & \sigma ∼N\left(1,\lambda \right) \end{array}$$

where $x_{i}$ represents the original signal sampling points, and $x_{i}^{\prime }$ represents the sampling points after noise injection. $\varepsilon ∼N\left(0,\lambda \right)$ denotes Gaussian noise that follows a normal distribution with a mean of 0 and a variance of λ. σ is the scaling factor, and σ∼N(1,λ) represents Gaussian noise with a mean of 1 and a variance of λ. λ is the variance, and the larger the value of λ, the greater the difference between the training and test sets.

To ensure a fair comparison across models, all methods use exactly the same training configuration: the AdamW optimizer is employed with an initial learning rate of 0.001 and a weight decay of 0.01. The learning rate scheduling adopts the ReduceLROnPlateau strategy, where the learning rate is reduced to 10% of its current value if the validation loss does not improve for 5 consecutive epochs. The minimum learning rate is limited to 0.00001. The batch size is fixed at 32, and the model is trained for a total of 100 epochs.

To evaluate our model, we adopted accuracy, complexity, recall, precision, and F1-score as key performance metrics [48,49]. To ensure robustness, we conducted five consecutive experiments and computed the aforementioned metrics for each run, reporting their averaged values. We followed an iterative train–validation strategy: after each epoch, we evaluated model performance on the validation set; in the latter half of training, we saved the checkpoint with the highest validation accuracy and used it for the final test-set evaluation. Note that no cross-validation was performed.

The experimental environment is configured as follows: PyTorch 1.12.0, CPU Intel Core i5-12600KF, and GPU NVIDIA GeForce GTX 4060 Ti.

### 4.2. Performance on the XJTU Dataset

**Figure 7:**
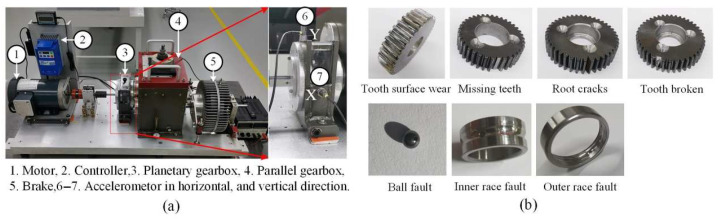
XJTU dataset: (**a**) experimental setup; (**b**) fault types.

The experiment uses a publicly available dataset collected from a planetary gearbox at Xi’an Jiaotong University [50], with the experimental setup shown in Figure 7a. The test rig consists of a motor, controller, planetary gearbox, parallel gearbox, brake, and accelerometers for collecting vibration signals in both horizontal and vertical directions.

In the experiment, vibration signals were synchronously collected in the axial and radial directions under the conditions of a constant motor speed of 1800 rpm and a sampling frequency of 20,480 Hz. The dataset covers four predefined fault modes for the planetary gearbox and four predefined fault modes for the bearings, totaling eight fault states. Additionally, signals from the healthy state were collected as a benchmark, resulting in nine state categories in total, as shown in Figure 7b. All vibration samples have a fixed length of 1024 data points, with a total of 10,800 samples. The detailed sample information is shown in Table 2. This partitioning ensures the training set contains enough samples for the model to fully learn each fault’s features, while retaining ample samples in the test set to yield a stable and reliable evaluation.

**Figure 8:**
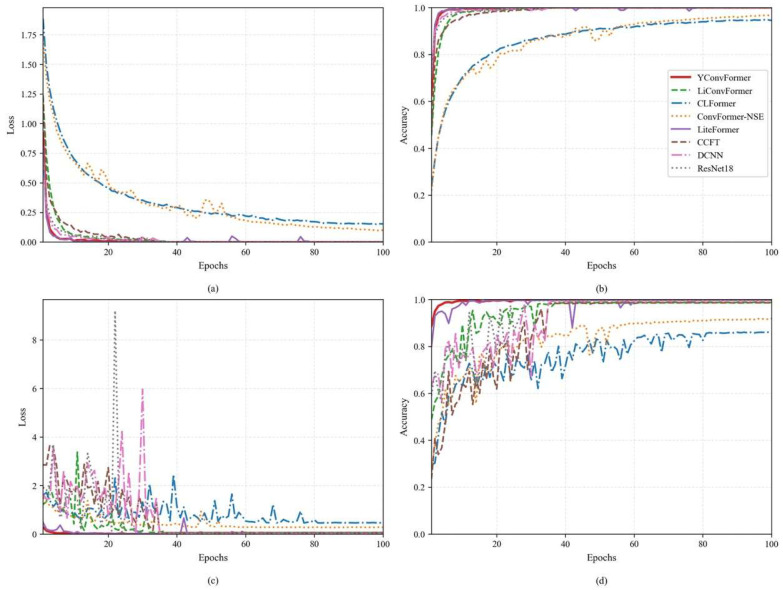
The loss and accuracy of each model: (**a**,**b**) training phase; (**c**,**d**) validation phase.

As shown in Figure 8, the average loss and accuracy curves during the training and validation phases for the ten experiments indicate the following: in the early stages of iteration, the loss and accuracy fluctuations on the training set for all methods were significantly smaller than those on the validation set. Subsequently, upon reaching 60 training epochs, all models exhibited stable low loss and high accuracy on both the training and validation datasets. Nevertheless, the validation accuracy of CLFormer and ConvFormer-NSE lagged noticeably behind the remaining six approaches. This phenomenon can be attributed to the feature dimension reduction strategy used by these two models, which led to the loss of high-frequency details in the vibration signals, thereby weakening the models’ ability to analyze multi-dimensional fault features. It is worth noting that although DCNN and CCFT eventually achieved ideal loss and accuracy in the test phase, their early curves exhibited severe oscillations. This is a typical manifestation of model over-parameterization, reflecting issues such as unstable gradients during training, low computational efficiency, and poor interpretability. In contrast, the proposed YConvFormer model, on the other hand, demonstrated average loss and accuracy fluctuations that were only slightly higher than LiteFormer. However, it exhibited the best stability and convergence during the validation phase, indicating that its parameter optimization trajectory consistently approached the global optimal solution. Ultimately, this low fluctuation characteristic confirms that the model effectively captures the spatiotemporal correlations of fault features, while also validating its outstanding generalization ability and noise robustness.

The comprehensive analysis in Table 3 indicates that YConvFormer achieves the optimal balance between noise robustness, computational efficiency, and diagnostic accuracy. Specifically, under strong noise interference (λ = 0.6), YConvFormer achieves an average accuracy of 87.93%, which is 6.52 percentage points higher than the best baseline, LiteFormer (81.41%). Compared to the traditional CNN model, ResNet18 (69.24%), it improves by 18.69 percentage points. Under low-noise conditions (λ = 0 and λ = 0.2), YConvFormer exhibits negligible variability with standard deviations of 0 and 0.200, respectively, surpassing all competing models. At higher noise levels, the standard deviation increases to 1.9545 at λ = 0.4 and to 2.5768 at λ = 0.6. However, the model’s pronounced accuracy advantage under these conditions firmly establishes YConvFormer as the most effective architecture overall.

Furthermore, YConvFormer achieves significant computational efficiency with only 0.604 M parameters (63.8% of LiteFormer) and 27.646 M FLOPs, representing an 88.1% reduction compared to DCNN. While CLFormer and ConvFormer-NSE exhibit lower complexity (0.143 M parameters and 6.270 M FLOPs, respectively), their accuracies under high noise drop to 65.96% and 60.58%, which are 21.97 and 27.35 percentage points lower than YConvFormer. These results demonstrate the effectiveness of the proposed architecture in achieving both robustness and lightweight design. Ultimately, the experimental data confirms that YConvFormer outperforms current advanced models in terms of noise adaptability, resource efficiency, and real-time performance, offering a more feasible, robust diagnostic solution for industrial scenarios.

**Figure 9:**
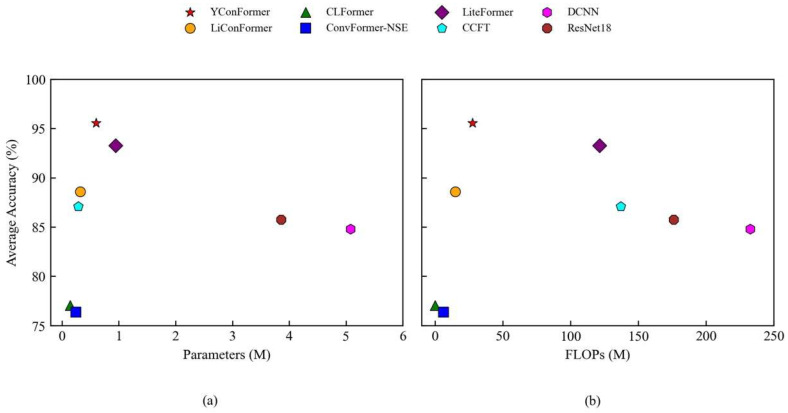
Average accuracy and model complexity: (**a**) parameters vs. accuracy; (**b**) FLOPs vs. accuracy.

Figure 9 provides a comprehensive comparison of diagnostic performance and computational complexity under various noise levels. The proposed YConvFormer exhibits remarkable noise robustness while achieving superior computational efficiency. Remarkably, with only 0.604 M parameters and 27.646 M FLOPs, it maintains an average accuracy of 87.93% under strong noise conditions (λ = 0.6), significantly outperforming all comparison models. Compared to the second-best performer, LiteFormer (81.41% accuracy), YConvFormer achieves a 6.52 percentage point gain, while reducing the parameter count by 36.2% and FLOPs by 77.2%. When compared with the lightweight CLFormer, it delivers a 21.97% higher accuracy with only 0.461 M additional parameters. Furthermore, relative to the conventional CNN model ResNet18, YConvFormer compresses the parameter size and computational cost by 84.3% while improving accuracy by 18.7 percentage points. These results affirm the model’s ability to balance robustness, accuracy, and efficiency, making it well-suited for real-time industrial fault diagnosis under noisy conditions.

**Figure 10:**
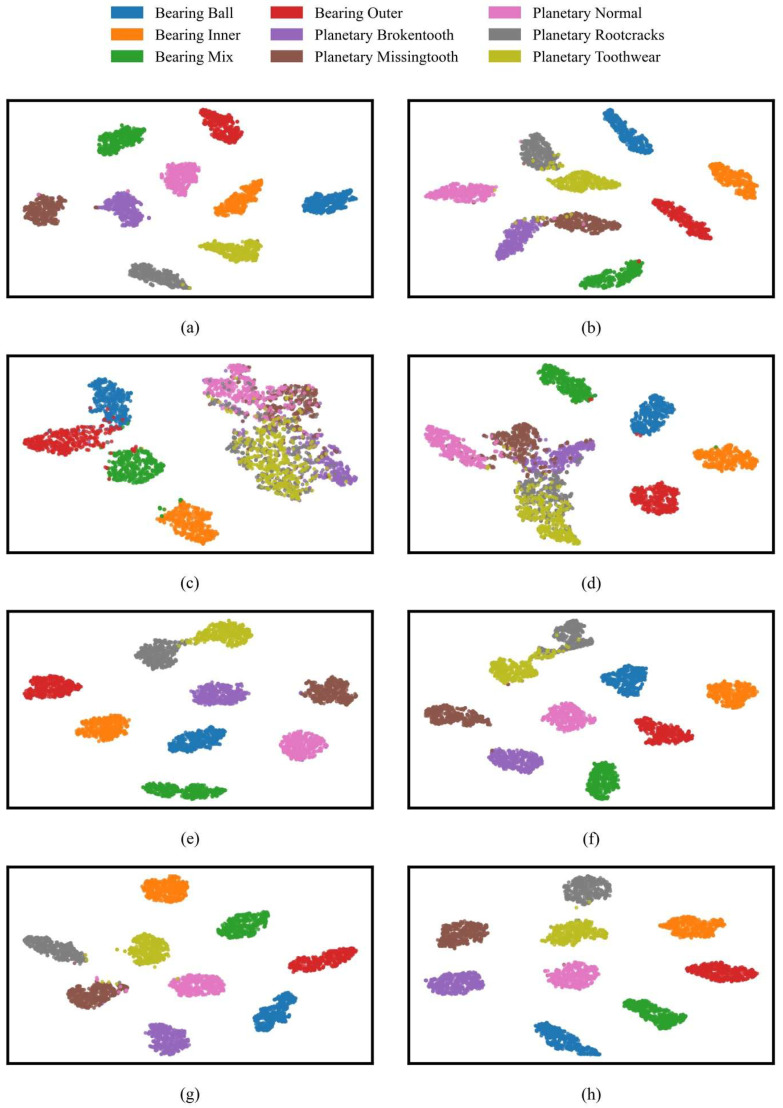
t-SNE visualization on the XJTU dataset: (**a**) YConvFormer; (**b**) LiConvFormer; (**c**) CLFormer; (**d**) ConvFormer-NSE; (**e**) LiteFormer; (**f**) CCFT; (**g**) DCNN; and (**h**) ResNet18.

Figure 10 illustrates the two-dimensional t-SNE distribution of feature representations produced by eight different models under noise-free conditions (λ = 0). Most notably, the features extracted by YConvFormer show exceptional discriminability. Samples of various fault types form compact and distinct clusters in the 2D space, with high intra-class sample density and clear boundaries between different fault states, providing strong evidence that YConvFormer can accurately capture fine-grained feature differences between fault and normal states. The second-best performing LiteFormer is able to cluster most of the healthy state samples, but some fault categories, such as root crack and tooth wear, show overlapping edge samples, with significantly weaker feature separability compared to YConvFormer. Meanwhile, lightweight CNN–Transformer models such as CLFormer and ConvFormer-NSE exhibit significant mixing and dispersal in their feature distribution, with large overlaps in the feature clusters for root crack and tooth wear, reflecting feature information loss due to excessive dimension compression. Traditional deep models like ResNet18 show unclear boundaries in the normal state clusters, with scattered normal state samples and indistinct separation from the fault clusters. This observation aligns with the sharp drop in their average accuracy to 68.41% and 69.24% under high noise conditions, revealing their insufficient noise resistance at the feature layer level. Importantly, these feature space distribution differences are closely aligned with model performance trends: YConvFormer, while maintaining a lightweight architecture, strengthens the separability of features between fault and normal states through an efficient feature encoding mechanism. As a result, it maintains an average accuracy of 87.93% at λ = 0.6, far surpassing other models. The t-SNE visualization further clarifies the underlying mechanism that makes YConvFormer superior in classification accuracy and noise robustness.

**Figure 11:**
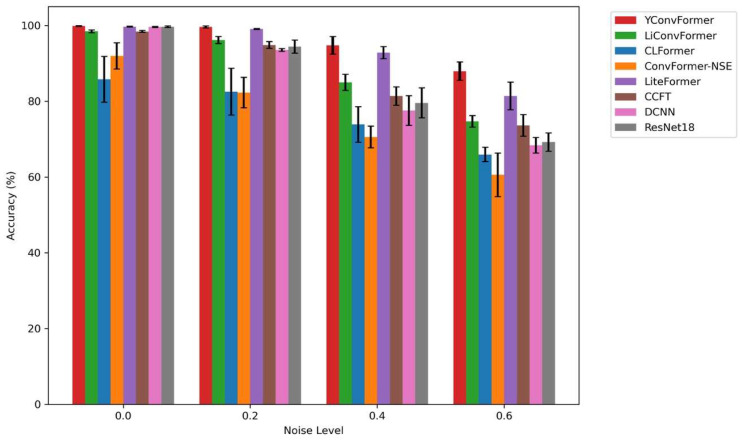
Average accuracy of each model at different λ levels on the XJTU dataset.

Figure 11 intuitively presents the diagnostic accuracy and fluctuation characteristics of eight models across four noise levels. YConvFormer demonstrates both leading accuracy and exceptional stability across the entire noise range, exhibiting a dual advantage: At λ = 0, its accuracy approaches 100%, on par with LiteFormer and ResNet18, with the shortest error bars, reflecting the stability of feature extraction. At λ = 0.2, the accuracy of CLFormer and ConvFormer-NSE decreases significantly, while YConvFormer maintains a high accuracy of around 99.60%, outperforming the next best model, LiteFormer, by approximately 0.51%. At λ = 0.4, YConvFormer achieves an accuracy of 94.77%, far surpassing lightweight models like CLFormer (73.88%) and ConvFormer-NSE (70.58%), and improving by 15.19 percentage points over the traditional strong baseline ResNet18 (79.58%). Most impressively, at λ = 0.6, YConvFormer maintains an accuracy of 87.93%, significantly outperforming LiteFormer (81.41%) and surpassing ResNet18 by 18.69%. Additionally, its error bars are noticeably shorter than those of the other models, further validating the effectiveness of its noise-robust feature enhancement mechanism. In contrast, while LiteFormer achieves similar accuracy to YConvFormer at low noise levels, its error bars increase sharply as noise increases. At λ = 0.6, the fluctuation range expands significantly, reflecting its insufficient noise stability. Overall, the bar chart quantifies and verifies the superiority of the YConvFormer model across three dimensions: accuracy, noise robustness decay rate, and result stability.

Table 4 shows that YConvFormer consistently outperforms other models, particularly in terms of precision, recall, and F1-score. Under low noise conditions (λ = 0 and λ = 0.2), YConvFormer achieves a perfect precision, recall, and F1-score of 0.9986. Even with increasing noise (λ = 0.4 and λ = 0.6), its performance remains strong, with F1-scores of 0.9487 and 0.8839, respectively.

In contrast, other models like LiConvFormer show a significant decline under high noise. At λ = 0.6, its F1-score drops to 0.7461, much lower than YConvFormer. DCNN and LiteFormer, while maintaining relatively higher performance, also experience notable drops as noise increases. For DCNN, the F1-score decreases from 0.9959 at λ = 0 to 0.6936 at λ = 0.6, while LiteFormer drops from 0.9971 at λ = 0 to 0.8164 at λ = 0.6. CLFormer and ConvFormer-NSE show even larger declines, with F1-scores of 0.6550 and 0.6094 at λ = 0.6, respectively, highlighting their lower noise resilience. Overall, YConvFormer’s ability to maintain high performance under increasing noise levels makes it the most effective model in terms of noise robustness and diagnostic accuracy.

**Figure 12:**
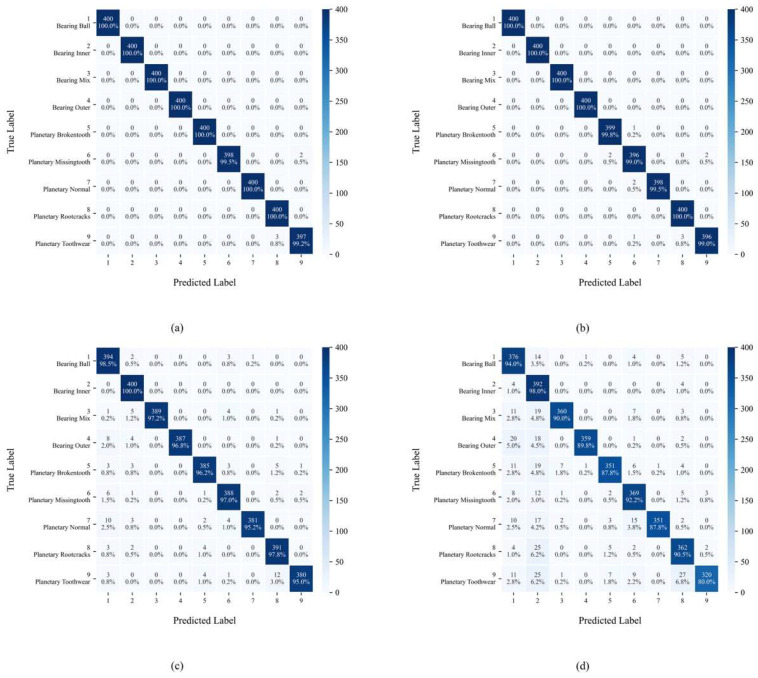
YConvFormer’s confusion matrix at different λ levels on the XJTU dataset: (**a**) λ = 0; (**b**) λ = 0.2; (**c**) λ = 0.4; and (**d**) λ = 0.6.

Figure 12 displays the confusion matrix distribution of YConvFormer at four different noise levels. The size and color depth of each matrix element reflect the frequency of predicted categories, while the values along the diagonal represent the classification accuracy of each true class. In Figure 12a, except for classes 6 and 9, all other classes achieve 100% classification accuracy, with only a very small number of samples being misclassified. This confirms the model’s exceptional ability to distinguish features in pure signals. In Figure 12b, although the diagonal values slightly decrease for each true class, it is significant that the number of misclassifications remains in single digits, and the errors are concentrated in adjacent semantic categories, such as Brokentooth and Missingtooth, indicating that noise has only a minor impact on the feature discrimination of edge samples. In Figure 12c, the diagonal values further decrease, and misclassifications become more dispersed across categories. However, the predicted distributions for each true class still cluster around the diagonal, indicating that the model is able to anchor core feature dimensions, even under moderate noise levels, maintaining correct classification for most samples. In Figure 12d, while the proportion of diagonal values significantly decreases and misclassifications across categories increase, compared to the average accuracy of other models under the same noise levels in Table 3, YConvFormer still maintains the highest diagonal proportion. Moreover, the misclassified samples do not exhibit a “random confusion across all categories” disorderly pattern, confirming that its noise-robust mechanism preserves key discriminative information and constrains the semantic validity of misclassified samples even under extreme noise conditions. In summary, the confusion matrix reveals YConvFormer’s highly robust classification characteristics under various noise levels from three dimensions: intra-class cohesion, cross-class confusion patterns, and performance degradation trends under increasing noise. This provides fine-grained feature-level evidence of its reliability in complex industrial noise environments.

### 4.3. Performance on SEU Dataset

**Figure 13:**
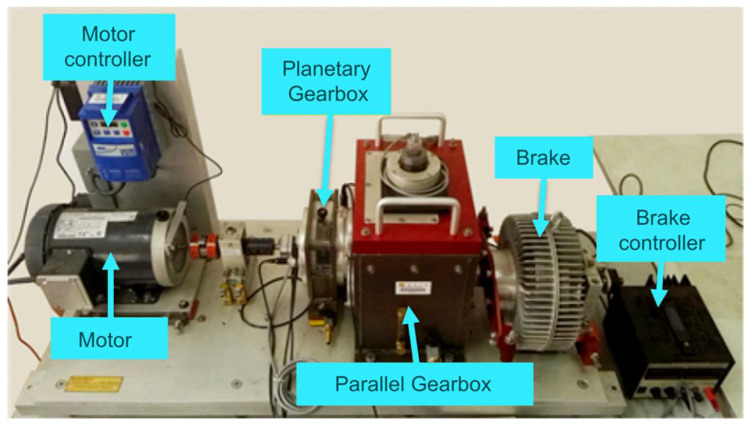
SEU dataset collection platform.

The second dataset used for model validation is provided by Southeast University (SEU) [51]. The experimental platform is shown in Figure 13, consisting mainly of a motor, motor controller, planetary gearbox, parallel gearbox, brake, and brake controller. The SEU gearbox dataset was collected using a drivetrain dynamics simulator (DDS). The experiment was conducted under two operating conditions: the speed–load configuration (RS-LC) was set to 20 Hz-0 V and 30 Hz-2 V, with load settings of 0 V and 2 V. Vibration signals were collected for eight different gear faults, four bearing faults, one normal gear state, and one normal bearing state. Vibration signals from eight channels were recorded, and the second channel’s vibration signal was input into the model. The dataset includes 10 types of data.

In this paper, the SEU gearbox dataset is preprocessed, and data with a load setting of 20 Hz–0 V is selected for the experiment. The dataset is first divided into training, validation, and test sets, with no overlap between them. Vibration signals are truncated using a sliding window approach without overlap, and each data sample contains 1024 points. All samples in the training set are used for training, half of the samples in the validation set are randomly selected for validation, and half of the samples in the test set are randomly selected for testing. Additionally, noise is added to the test set, as indicated in formula 12. Table 5 shows the dataset division. This partitioning ensures the training set provides ample samples for the model to learn robust fault representations, while reserving 25% each for validation and testing to support reliable hyperparameter tuning and unbiased performance evaluation.

Table 6 reaffirms the findings of the first experiment, where YConvFormer consistently outperforms competing models in terms of noise robustness. While its accuracy is slightly lower than LiteFormer and ResNet18 at low noise (λ = 0), it maintains a significant lead at higher noise levels. YConvFormer’s performance under strong noise (λ = 0.6) is notably better than models such as LiteFormer, DCNN, and ResNet18, confirming its superiority in feature extraction and noise resilience. Despite a relatively higher standard deviation of 2.5768 at λ = 0.6 compared to its peers, YConvFormer’s markedly superior accuracy under these conditions confirms it as the overall best-performing model.

**Figure 14:**
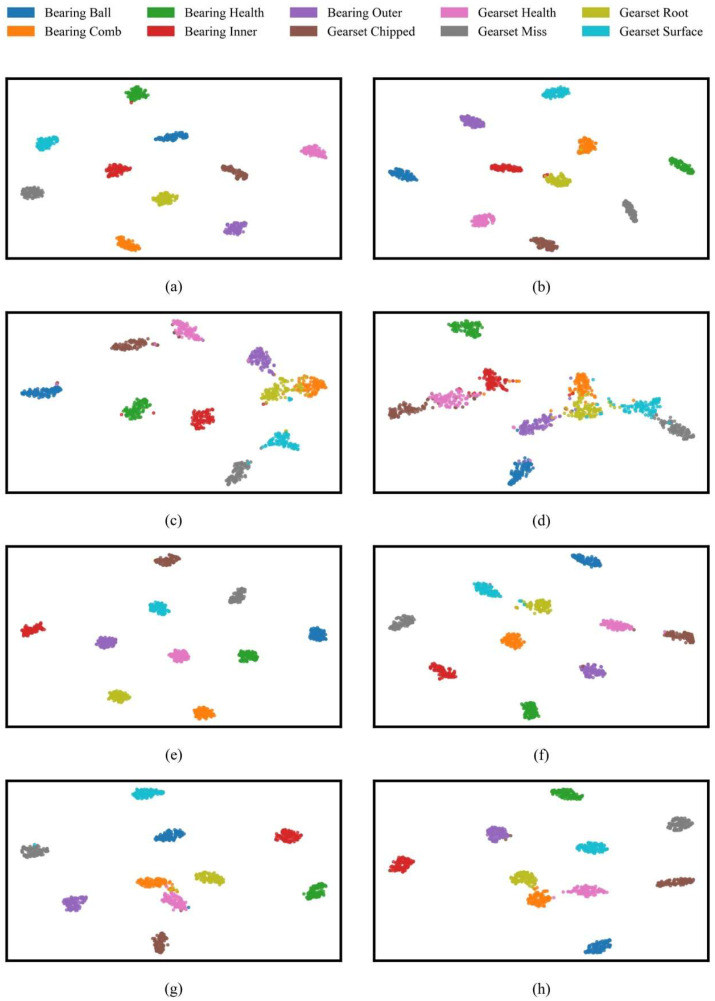
t-SNE visualization on SEU dataset: (**a**) YConvFormer; (**b**) LiConvFormer; (**c**) CLFormer; (**d**) ConvFormer-NSE; (**e**) LiteFormer; (**f**) CCFT; (**g**) DCNN; and (**h**) ResNet18.

Figure 14 shows that the t-SNE analysis in this experiment mirrors the previous findings, where YConvFormer exhibits clear, compact feature clusters with minimal overlap. This highlights its exceptional ability to distinguish between fault and normal states, even under noisy conditions. The performance of LiteFormer is still strong under low noise but shows increasing feature overlap as noise levels rise, further emphasizing YConvFormer’s robust discriminability.

**Figure 15:**
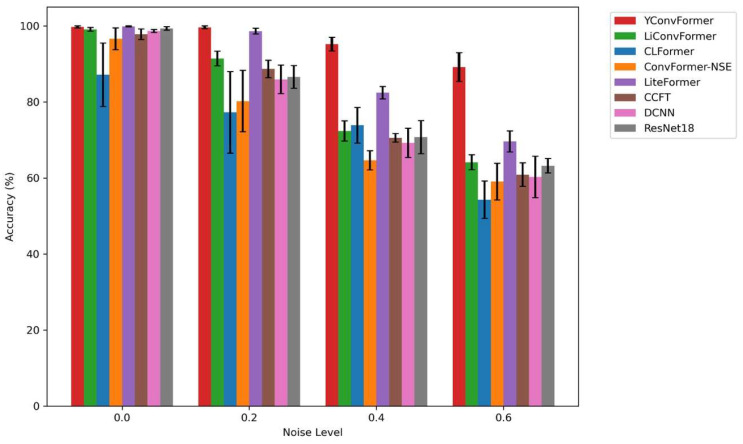
Average accuracy of each model at different λ levels on SEU dataset.

The bar chart in Figure 15 shows that YConvFormer again demonstrates its dual advantage of accuracy and stability. At higher noise levels (λ = 0.4 and λ = 0.6), it maintains a much smaller drop in accuracy compared to other models, particularly those like CLFormer and LiConvFormer, which show sharp declines. These results corroborate the findings from the first experiment, where YConvFormer’s robust feature extraction mechanism ensures minimal performance degradation even in challenging conditions.

Table 7 confirms the findings of the experiment on the XJTU dataset: YConvFormer consistently leads in noise robustness. At low noise (λ = 0), it records precision = 0.9975, recall = 0.9974, and F1-score = 0.9974—just behind LiteFormer (0.9986) and ahead of ResNet18 (0.9935). As noise rises, YConvFormer retains its advantage. Under strong noise (λ = 0.6), it achieves precision = 0.9174, recall = 0.8962, and F1-score = 0.8984, surpassing both LiteFormer and ResNet18 and demonstrating superior stability in noisy conditions. Although its standard deviation at λ = 0.6 is higher than some peers, its accuracy secures its status as the optimal model overall.

**Figure 16:**
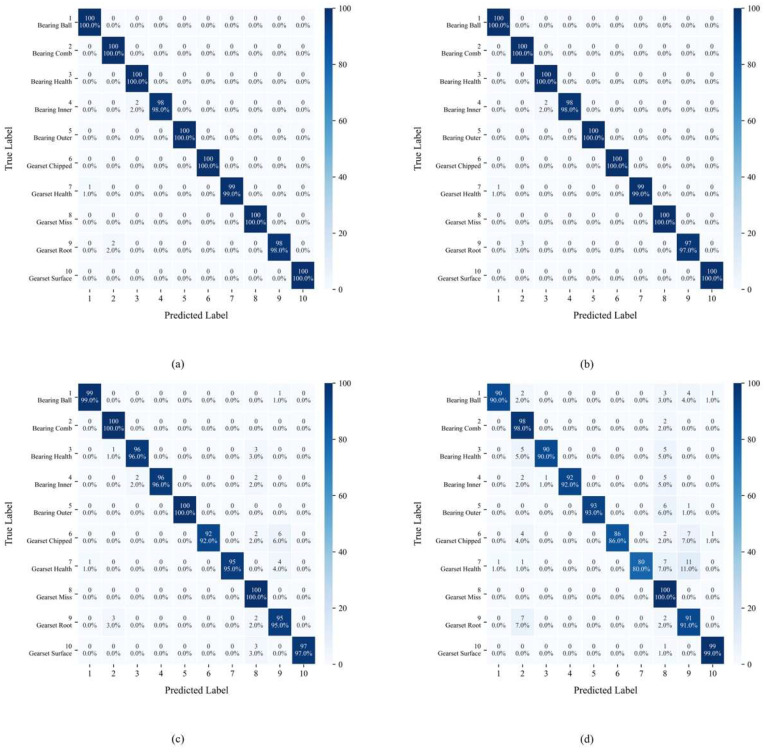
YConvFormer’s confusion matrix at different λ levels on SEU dataset: (**a**) λ = 0; (**b**) λ = 0.2; (**c**) λ = 0.4; and (**d**) λ = 0.6.

Figure 16 presents the confusion matrix that further supports the conclusions drawn in the first experiment. Even under extreme noise, YConvFormer shows superior ability in maintaining high intra-class cohesion and preventing random misclassification. The misclassifications primarily occur between semantically similar classes, which is indicative of the model’s noise-robust feature extraction and classification capability.

### 4.4. Ablation Experiment

This section validates the effectiveness of the proposed modules on both the SEU and XJTU datasets. The data partitioning follows the same method as described earlier.

Table 8 presents the fault diagnosis performance of time-domain (TD), frequency-domain (FD), and time–frequency combined (TFE) inputs with the AEBA module on both the XJTU and SEU datasets. Most significantly, TFE outperforms all other input methods at every noise level across both datasets, demonstrating the stability of time–frequency fusion. Breaking down the results, TD achieves high accuracy at λ = 0, but as noise increases, its accuracy drops significantly at λ = 0.6. Specifically, on the XJTU dataset, the accuracy decreases to 0.7834, and on the SEU dataset, it drops to 0.6566. Meanwhile, the standard deviation for TD rises from 0.1790 to 3.3128 on XJTU (and from 0.3000 to 1.5969 on SEU), indicating increasing inconsistency under noise. This indicates that time-domain signals are highly susceptible to noise interference, and the lack of frequency-domain information leads to performance degradation in high-noise conditions. Turning to FD input, although more robust to noise than TD, it consistently has lower accuracy than TFE due to the absence of time-domain transient details. FD’s standard deviation also grows from 0.1000 to 3.3151 on XJTU (and from 0.2301 to 2.5315 on SEU), reflecting similar stability issues at high noise levels. By contrast, TFE effectively combines the transient pulse features from the time domain with the energy distribution features from the frequency domain, effectively enhancing fault information through complementary feature fusion. Notably, TFE maintains the lowest standard deviations across all noise levels (0.0000 → 2.5768 on XJTU and 0.1854 → 3.0800 on SEU); on the SEU dataset, although TFE’s standard deviation at λ = 0.6 (3.0800) exceeds that of the next best model (2.5315), its higher accuracy (0.8920 vs. 0.8708) confirms it as the best-performing method. In all noise scenarios across both datasets, TFE consistently leads, strongly validating the necessity and effectiveness of time–frequency joint inputs for gearbox fault diagnosis.

**Figure 17:**
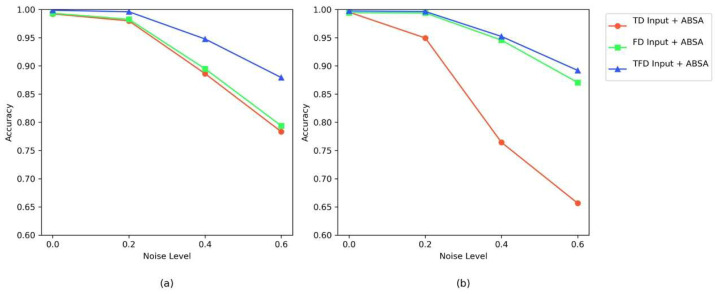
Anti-noise accuracy curves of different input types on dual datasets: (**a**) XJTU dataset; (**b**) SEU dataset.

Figure 17 presents the average accuracy curves for different inputs (time-domain TD, frequency-domain FD, time–frequency combined TFE) with the AEBA module on two datasets. Time–frequency combined (TFE) input shows the strongest noise robustness across all noise levels: In Figure 17a, under low noise conditions, the accuracy of TFE is comparable to that of TD and FD. However, in high-noise conditions, TFE experiences the smallest accuracy drop. In this figure, TFE drops from approximately 0.99 to 0.87, which is significantly smaller than the drop in TD (from 0.99 to 0.78) and FD (from 0.99 to 0.79). In Figure 17b, at λ = 0.6, TFE still maintains an accuracy of about 0.88, while TD drops to just 0.65. The diagnostic efficiency of FD is comparable to TFE, far outperforming TD. This is because the SEU dataset is more sensitive to noise, and frequency-domain input retains fault information better, showing better noise resistance [52]. This aligns with the experimental results where YConvFormer outperforms LiteFormer by 19.58% under strong noise conditions, demonstrating that incorporating frequency-domain information significantly enhances accuracy. Each subplot confirms that time–frequency joint input preserves complementary information from both time-domain transient features and frequency-domain energy distribution, enhancing fault discriminability under noise. The consistent leading performance of TFE across both datasets further demonstrates the critical gain in noise robustness provided by the time–frequency fusion strategy, offering empirical support for the effectiveness of the AEBA module in terms of the input domain.

Table 9 presents the fault diagnosis accuracy of TFE input combined with different attention mechanisms (MSA, BSA, and AEBA) on the XJTU and SEU datasets. TFE Input + AEBA demonstrates a dual advantage of robustness across datasets and adaptability of the attention mechanism, showing superior performance from λ = 0 to λ = 0.6. Specifically, in low-noise scenarios (λ = 0, λ = 0.2), the accuracies and standard deviations of MSA, BSA, and AEBA are similar. However, in high-noise scenarios (λ = 0.4, λ = 0.6), AEBA shows a significant advantage over both MSA and BSA; although its standard deviation remains at an intermediate level, its markedly higher accuracy supports its identification as the best-performing method.

**Figure 18:**
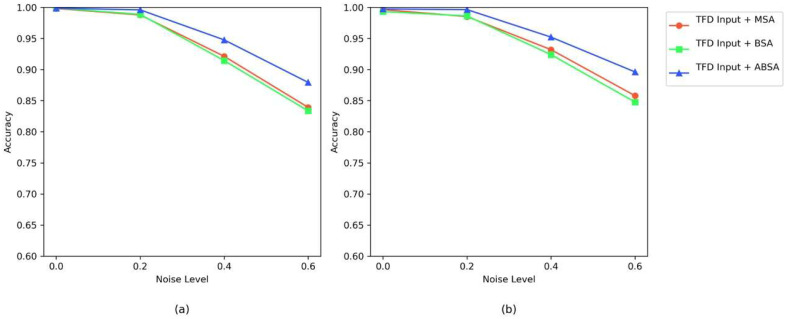
Anti-noise accuracy curves of different attention mechanisms with TFE input on dual datasets: (**a**) XJYU dataset; (**b**) SEU dataset.

Figure 18 displays the anti-noise accuracy curves for TFE input combined with different attention mechanisms (MSA, BSA, and AEBA) across dual datasets. Most notably, the model incorporating Axial Decomposition (AEBA) demonstrates the best noise resistance across all noise levels. The axial decomposition feature of AEBA allows the decoupling of multi-dimensional dependencies in time–frequency domain features, preventing single-dimensional attention from overlooking critical information. Furthermore, its design to expand the receptive field strengthens cross-scale feature associations, enabling the model to reliably capture fault time–frequency coupling patterns even under noise interference. Ultimately, the consistent superior performance of AEBA across both datasets validates the enhancement in attention-based noise resistance brought by axial decomposition and receptive field optimization.

## 5. Conclusions

This paper addresses the challenge of balancing lightweight design and noise robustness in gearbox fault diagnosis by proposing the YConvFormer model. Specifically, the core innovations of this model are as follows: The TFE input fuses time-domain information and frequency-domain information at the input level, and performs channel weighting via ECA to suppress noise interference. The design of AEBA, which utilizes spatial axial modeling to capture long-range temporal dependencies, expands the receptive field of shock features and strengthens cross-frequency harmonic interactions via channel axial convolution. Notably, this approach reduces computation by avoiding matrix multiplications and multi-dimensional power operations. A lightweight robust collaborative architecture, combining shallow feature processing with AEBA-convolution-based global–local modeling, ultimately achieves significant compression with only 0.604 M parameters and 27.646 M FLOPs. In experiments on the XJTU and SEU datasets, the model achieves average accuracies of 87.93% and 89.20% under strong noise conditions (λ = 0.6), outperforming the best baseline model, LiteFormer, by 6.52% and 19.58%, respectively. Moreover, ablation experiments confirm the significant contributions of TFE and AEBA to noise robustness. The model achieves the optimal balance in accuracy, noise resistance, and computational efficiency compared to seven benchmark models.

While the model achieves an optimal balance between accuracy, noise resistance, and computational efficiency, certain limitations remain. The model’s performance may still be affected by extremely low signal-to-noise ratios or highly variable operating conditions that were not fully explored in this study. Furthermore, its application in real-time fault diagnosis, especially in edge devices with limited resources, could present additional challenges related to further computational constraints and hardware compatibility.

Future research will focus on the deployment of the YConvFormer model in edge computing environments to assess its real-time diagnostic capabilities. Additionally, exploring its adaptability across different industrial scenarios and further improving its noise resilience under extreme conditions could enhance the model’s robustness and practical applicability. In future work, we will conduct data collection and validation on a custom-built platform to simulate real-world operating conditions, enhancing the robustness of the model under actual industrial environments.

## Figures and Tables

**Table 1:** Core parameters and signal shapes of the model.

Layer	Blocks	Parameters	Signal Shape
Input Layer	/	/	[B, 2n, 1024]
Time Branch	AvgPool1d	kernel = 2, stride = 2	[B, n, 512]
	ConvBNReLU	in = 2, out = 32, k = 15, s = 2	[B, 32, 256]
Freq Branch	AvgPool1d	kernel = 2, stride = 2	[B, n, 512]
	ConvBNReLU	in = 2, out = 32, k = 15, s = 2	[B, 32, 256]
ECA Layer	Conv1d	in = 64, out = 64, k = 5	[B, 64, 256]
Deep Feature Extraction Layer1	Embedding	in = 64, out = 64, stride = 2	[B, 64, 128]
	AEBA	dim = 64, k_s_ = 15, k_c_ = 1	[B, 64, 128]
	FFN	r = 4	[B, 64, 128]
Deep Feature Extraction Layer2	Embedding	in = 128, out = 128, stride = 2	[B, 128, 64]
	AEBA	dim = 128, ks = 15,kc = 1	[B, 128, 64]
	FFN	r = 4	[B, 128, 64]
Deep Feature Extraction Layer3	Embedding	in = 256, out = 256, stride = 2	[B, 256, 32]
	AEBA	dim = 256, ks = 15, kc = 1	[B, 256, 32]
	FFN	r = 4	[B, 256, 32]
Output Layer	AvgPool1d	K = 32, s = 32	[B, 256]
	Linear	d = N	[B, N]

The input signal length is fixed at 1024 data points, and B denotes the batch size, n denotes the number of channels, and k_s_ denotes the spatial axial convolution kernel size, which controls the receptive field along the temporal dimension in the AEBA module; k_c_ refers to the channel axial convolution kernel size, which determines the feature interaction scope across channels; N represents the number of output fault categories.

**Table 2:** Sample numbers and data partitioning on the XJTU dataset.

Type of Fault	Fault Category	Train Data	Valid Data	Test Data	Total
Bearing Ball	1	500	300	400	1200
Bearing Inner	2	500	300	400	1200
Bearing Mix	3	500	300	400	1200
Bearing Outer	4	500	300	400	1200
Planetary Brokentooth	5	500	300	400	1200
Planetary Missingtooth	6	500	300	400	1200
Planetary Normal	7	500	300	400	1200
Planetary Rootcracks	8	500	300	400	1200
Planetary Toothwear	9	500	300	400	1200

**Table 3:** Model accuracy and complexity on the XJTU dataset.

Model	Noise Level	Mean Accuracy	Max Accuracy	Standard Deviations	Params (M)	Flops (M)	Inference Time(s)
YConvFormer	0.0	0.9986	0.9994	0	0.604	27.646	0.697
	0.2	0.9960	0.9986	0.2000			
	0.4	0.9477	0.9708	1.9545			
	0.6	0.8793	0.9036	2.5768			
LiConvFormer	0.0	0.9846	0.9883	0.2828	0.322	14.976	0.518
	0.2	0.9616	0.9711	0.6164			
	0.4	0.8501	0.8714	1.4387			
	0.6	0.7468	0.7622	1.2083			
CLFormer	0.0	0.8580	0.9186	5.0110	0.143	0.005	0.496
	0.2	0.8253	0.8872	4.5305			
	0.4	0.7388	0.7858	2.7240			
	0.6	0.6596	0.6783	1.5000			
ConvFormer-NSE	0.0	0.9198	0.9542	2.3043	0.245	6.270	0.721
	0.2	0.8230	0.8633	3.9205			
	0.4	0.7058	0.7344	3.2638			
	0.6	0.6058	0.6636	4.1231			
LiteFormer	0.0	0.9971	0.9983	0.1000	0.946	121.276	0.536
	0.2	0.9909	0.9922	0.1414			
	0.4	0.9284	0.9442	0.9644			
	0.6	0.8141	0.8508	2.1772			
CCFT	0.0	0.9844	0.9869	0.2236	0.288	137.076	2.391
	0.2	0.9486	0.9575	0.8718			
	0.4	0.8139	0.8381	2.5690			
	0.6	0.7363	0.7647	2.1284			
DCNN	0.0	0.9959	0.9972	0.1000	5.077	232.543	0.931
	0.2	0.9356	0.9392	0.4123			
	0.4	0.7754	0.8147	2.2472			
	0.6	0.6841	0.7047	1.6462			
ResNet18	0.0	0.9969	0.9989	0.1414	3.854	175.920	0.7533
	0.2	0.9444	0.9617	1.2166			
	0.4	0.7958	0.8353	2.8775			
	0.6	0.6924	0.7167	1.6703			

**Table 4:** Model Performance on the XJTU dataset: average precision, average recall, and average F1-score.

Model	Noise Level	Average Precision	Average Recall	Average F1-Score
YConvFormer	0.0	0.9986	0.9986	0.9986
	0.2	0.9960	0.9960	0.9960
	0.4	0.9522	0.9477	0.9487
	0.6	0.9001	0.8793	0.8839
LiConvFormer	0.0	0.9847	0.9845	0.9845
	0.2	0.9621	0.9609	0.9609
	0.4	0.8698	0.8504	0.8489
	0.6	0.7939	0.7464	0.7461
CLFormer	0.0	0.8585	0.8580	0.8579
	0.2	0.8253	0.8253	0.8238
	0.4	0.7500	0.7388	0.7342
	0.6	0.6895	0.6595	0.6550
ConvFormer-NSE	0.0	0.9209	0.9198	0.9200
	0.2	0.8357	0.8230	0.8195
	0.4	0.7432	0.7058	0.7045
	0.6	0.6688	0.6058	0.6094
LiteFormer	0.0	0.9971	0.9971	0.9971
	0.2	0.9909	0.9909	0.9909
	0.4	0.9348	0.9284	0.9283
	0.6	0.8631	0.8140	0.8164
CCFT	0.0	0.9844	0.9844	0.9844
	0.2	0.9516	0.9486	0.9486
	0.4	0.8518	0.8146	0.8146
	0.6	0.7963	0.7363	0.7352
DCNN	0.0	0.9960	0.9959	0.9959
	0.2	0.9441	0.9360	0.9340
	0.4	0.8605	0.7754	0.7823
	0.6	0.8059	0.6840	0.6936
ResNet18	0.0	0.9970	0.9970	0.9970
	0.2	0.9520	0.9444	0.9427
	0.4	0.8537	0.7959	0.7973
	0.6	0.7977	0.6925	0.6988

**Table 5:** Sample numbers and data partitioning on SEU dataset.

Type of Fault	Fault Category	Train Data	Valid Data	Test Data	Total
Bearing Ball	1	200	100	100	400
Bearing Comb	2	200	100	100	400
Bearing Health	3	200	100	100	400
Bearing Inner	4	200	100	100	400
Bearing Outer	5	200	100	100	400
Gearset Chipped	6	200	100	100	400
Gearset Health	7	200	100	100	400
Gearset Miss	8	200	100	100	400
Gearset Root	9	200	100	100	400
Gearset Surface	10	200	100	100	400

**Table 6:** Model accuracy and complexity on SEU dataset.

Model	Noise Level	Mean Accuracy	Max Accuracy	Standard Deviations	Params (M)	Flops (M)	Inference Time(s)
YConvFormer	0.0	0.9974	1.0000	0.1854	0.605	27.646	0.166
	0.2	0.9964	1.0000	0.2059			
	0.4	0.9522	0.9700	2.0410			
	0.6	0.8920	0.9300	3.0800			
LiConvFormer	0.0	0.9910	0.9960	0.3286	0.323	14.846	0.113
	0.2	0.9146	0.9340	1.3749			
	0.4	0.7236	0.7500	2.1676			
	0.6	0.6414	0.6610	1.2909			
CLFormer	0.0	0.8716	0.9550	6.1245	0.133	0.005	0.142
	0.2	0.7726	0.8800	9.4432			
	0.4	0.7388	0.7858	4.7189			
	0.6	0.5430	0.5920	3.1898			
ConvFormer-NSE	0.0	0.9664	0.9950	2.7156	0.245	6.221	0.200
	0.2	0.8024	0.8830	5.6710			
	0.4	0.6466	0.6720	2.3636			
	0.6	0.5908	0.6390	3.9059			
LiteFormer	0.0	0.9986	1.0000	0.1000	0.945	121.145	0.133
	0.2	0.9864	0.9940	0.3873			
	0.4	0.8246	0.8410	1.4249			
	0.6	0.6962	0.7240	1.8028			
CCFT	0.0	0.9780	0.9920	0.8185	0.288	137.076	0.633
	0.2	0.8869	0.9100	1.2530			
	0.4	0.7056	0.7170	0.7810			
	0.6	0.6090	0.6400	1.9468			
DCNN	0.0	0.9870	0.9910	0.3464	5.077	232.314	0.226
	0.2	0.8594	0.8970	2.0175			
	0.4	0.6926	0.7310	3.3941			
	0.6	0.6028	0.6570	3.4496			
ResNet18	0.0	0.9934	0.9980	0.2828	3.854	175.691	0.199
	0.2	0.8660	0.8960	1.7550			
	0.4	0.7076	0.7510	2.4558			
	0.6	0.6322	0.6510	1.6853			

**Table 7:** Model Performance on SEU dataset: average precision, average recall, and average F1-score.

Model	Noise Level	Precision	Recall	F1-Score
YConvFormer	0.0	0.9975	0.9974	0.9974
	0.2	0.9965	0.9964	0.996
	0.4	0.9575	0.9622	0.9529
	0.6	0.9174	0.8962	0.8984
LiConvFormer	0.0	0.9912	0.9910	0.9910
	0.2	0.9303	0.9146	0.9154
	0.4	0.8508	0.7236	0.7366
	0.6	0.8148	0.6414	0.6670
CLFormer	0.0	0.8862	0.8716	0.8718
	0.2	0.8151	0.7626	0.7686
	0.4	0.7540	0.6094	0.6316
	0.6	0.7341	0.5430	0.5747
ConvFormer-NSE	0.0	0.9681	0.9664	0.9668
	0.2	0.8415	0.8024	0.8031
	0.4	0.8138	0.6466	0.6763
	0.6	0.8027	0.5908	0.6294
LiteFormer	0.0	0.9986	0.9986	0.9986
	0.2	0.9869	0.9864	0.9863
	0.4	0.8779	0.8246	0.8294
	0.6	0.9935	0.9934	0.9934
CCFT	0.0	0.9786	0.9780	0.9780
	0.2	0.9032	0.8868	0.8846
	0.4	0.8243	0.7056	0.7215
	0.6	0.8013	0.6090	0.6409
DCNN	0.0	0.9873	0.9870	0.9870
	0.2	0.8917	0.8594	0.8561
	0.4	0.8325	0.6926	0.7055
	0.6	0.8131	0.6028	0.6389
ResNet18	0.0	0.9935	0.9934	0.9934
	0.2	0.8956	0.8660	0.8619
	0.4	0.8372	0.7076	0.7161
	0.6	0.8167	0.6322	0.6599

**Table 8:** Accuracy of different inputs on two datasets.

		XJYU			SEU		
Model	Noise Level	Mean Accuracy	Max Accuracy	Standard Deviations	Mean Accuracy	Max Accuracy	Standard Deviations
TD Input + AEBA	0.0	0. 9923	0.9947	0.1790	0.9952	0.9980	0.3000
	0.2	0.9798	0.9958	0.3656	0.9494	0.9670	1.4318
	0.4	0.8862	0.9267	3.0929	0.7644	0.7850	2.1955
	0.6	0.7834	0.8392	3.3128	0.6566	0.6810	1.5969
FD Input + AEBA	0.0	0.9933	0.9944	0.1000	0.9944	1.0000	0.2301
	0.2	0.9825	0.9875	0.4123	0.9932	1.0000	0.9249
	0.4	0.8949	0.9222	2.9682	0.9450	0.9680	1.1743
	0.6	0.7938	0.8275	3.3151	0.8708	0.9150	2.5315
TFE Input + AEBA	0.0	0.9986	0.9994	0	0.9974	1.0000	0.1854
	0.2	0.9960	0.9986	0.2000	0.9964	1.0000	0.2059
	0.4	0.9477	0.9708	1.9545	0.9522	0.9700	2.0410
	0.6	0.8793	0.9036	2.5768	0.8920	0.9300	3.0800

**Table 9:** Accuracy of different attention mechanisms on dual datasets.

		XJYU		SEU			
Model	Noise Level	Mean Accuracy	Max Accuracy	Standard Deviations	Mean Accuracy	Max Accuracy	Standard Deviations
TFE Input + MSA	0.0	0.9982	0.9992	0.1000	0.9964	0.9980	0.1318
	0.2	0.9878	0.9925	0.5196	0.9852	0.9910	0.7743
	0.4	0.9213	0.9447	1.8947	0.9328	0.9500	2.0800
	0.6	0.8392	0.8689	2.0712	0.8580	0.8840	2.3128
TFE Input + BSA	0.0	0.9989	0.9994	0.3636	0.9932	0.9950	0.2301
	0.2	0.9887	0.9908	0.4123	0.9862	0.9900	0.9249
	0.4	0.9144	0.9264	1.7874	0.9240	0.9330	2.1790
	0.6	0.8336	0.8411	2.7280	0.8478	0.8620	3.1617
TFE Input + AEBA	0.0	0.9986	0.9994	0	0.9974	1.0000	0.1854
	0.2	0.9960	0.9986	0.2000	0.9964	1.0000	0.2059
	0.4	0.9477	0.9708	1.9545	0.9522	0.9700	2.0410
	0.6	0.8793	0.9036	2.5768	0.8920	0.9300	3.0800

## Data Availability

The dataset used in this article can be obtained from the corresponding author upon request.

## Nomenclature

FFT	Fast Fourier Transform
ECA	Efficient Channel Attention
TFE	Time–Frequency ECA
MHA	Multi-Head Attention
AEBA	Axial Enhanced Broadcast Attention
TD	Time Domain
FD	Frequency Domain
